# Comprehensive Orthodontic Therapy and Retention Protocol in Freeman–Sheldon Syndrome: A Case Report

**DOI:** 10.7759/cureus.92684

**Published:** 2025-09-19

**Authors:** Sandhra Madhu, Maneesha Achuthan P K, Preethy S Nair, Akshay Puthenpurayil, Sapna Varma N K

**Affiliations:** 1 Orthodontics, Aline Dental Hospital, Kannur, IND; 2 Orthodontics and Dentofacial Orthopedics, Amrita School of Dentistry, Amrita Institute of Medical Sciences, Kochi, IND

**Keywords:** digital orthodontics, essix retainer, freeman-burian syndrome, freeman-sheldon syndrome (fss), limited mouth opening, orthodontics brackets

## Abstract

Freeman-Sheldon syndrome (FSS) is a rare congenital disorder with craniofacial and musculoskeletal abnormalities, presenting unique orthodontic challenges due to microstomia and restricted oral access. This case report describes the successful non-surgical orthodontic management of a 15-year-old girl with severe crowding using pre-adjusted edgewise appliances. Favorable alignment and functional occlusion were achieved, and a customized retention protocol combining Essix and fixed lingual retainers ensured stability.

## Introduction

Freeman-Sheldon syndrome (FSS), also known as “whistling face syndrome”/“Freeman-Burian syndrome," was first reported in 1938 by E. A. Freeman and J. H Sheldon. It is a set of congenital disorders characterised by arthrogyposis [1]. This rare genetic disorder is characterised by musculoskeletal deformities affecting the face, hands, and feet, due to which it was initially called “cranio-carpal-tarsal dystrophy." Individuals with this syndrome have microstomia, nasal speech, and impaired hearing. Camptodactyly, ulnar deviations, and club feet are other non-craniofacial features seen in such patients [2]. Although rare, some cases have also reported mental retardation due to the involvement of the central nervous system [3].

FSS is usually diagnosed at the time of birth, but studies have shown that it can be diagnosed during the gestation period using ultrasonography. The mutation of the gene MYH3 is responsible for the autosomal dominant, sometimes autosomal recessive inheritance pattern of the syndrome [2]. Histologic findings showed that the normal muscle fibers were replaced with white tendinous tissues and adipose tissue. This resulted in increased muscle stiffness and restriction of movement [1].

 An extensive literature search revealed limited literature describing the dental aspects of FSS. This case report describes the orthodontic management of a patient diagnosed with FSS, as well as the retention protocol required for maintaining the corrections achieved.

## Case presentation

A 15-year-old girl reported to the Department of Orthodontics with the chief complaint of severe crowding in the upper and lower anterior region. A genetic test was conducted, which confirmed the FSS. The patient also reported a positive family history. The typical characteristics seen in FSS, such as microstomia, ulnar deviation, and club foot, were observed on general physical examination. However, the patient showed no signs of mental retardation.

**Figure 1:**
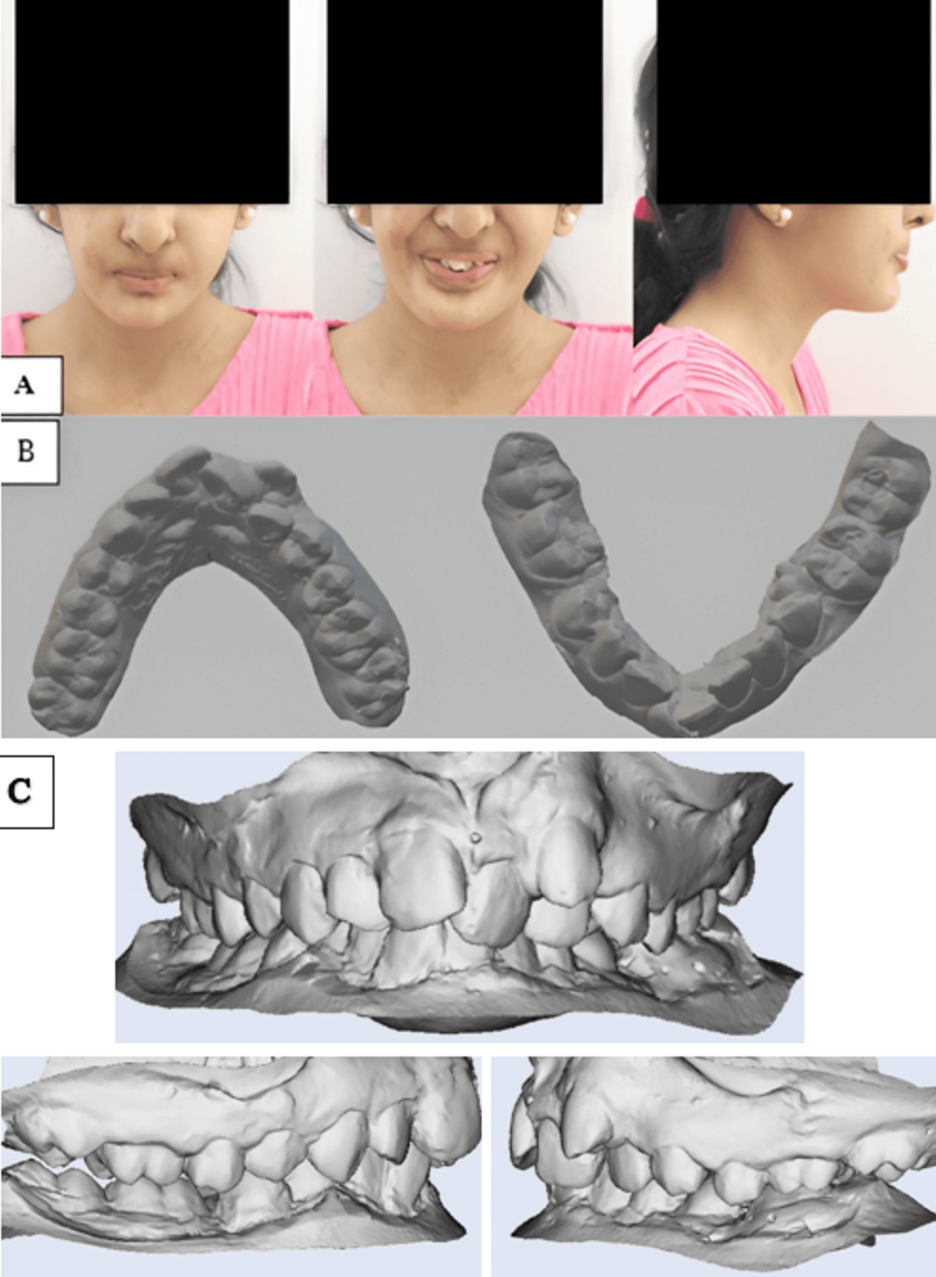
Pre-treatment records A. Pretreatment extraoral photos. B. Intraoral scan showing maxillary and mandibular arches. C. Intraoral scan showing right, frontal, and left occlusion.

Intraoral scans of the maxillary and mandibular arches were taken to record the patient’s pre-treatment dentition, considering the limited mouth opening (Figure 1). Sectional trays had to be used for taking the impression of each quadrant. Intraoral examination revealed an ectopically erupted canine in the maxillary arch, along with severe crowding in both the maxillary and mandibular arches. A bilateral Class I molar relationship was observed. Deep dental caries was noted in relation to tooth 46, likely associated with restricted mouth opening and compromised oral hygiene maintenance.

Craniofacial consultation recommended fibrotomy of the lips along with commisuroplasty before fixed appliance therapy to improve the mouth opening to facilitate bonding. The patient was not willing to undergo a commissuroplasty procedure, and therefore, a non-surgical orthodontic treatment plan was finalised.

**Figure 2:**
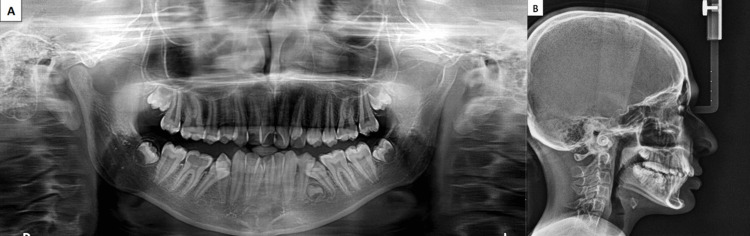
Pre-treatment radiographic records A. Preoperative orthopantomogram. B. Preoperative lateral cephalogram.

OPG revealed supernumerary tooth bud between 34 and 35, tooth buds 18, 28, 38, and 48, and dilaceration at 35. Cephalometric analysis revealed a Class I skeletal base with orthognathic maxilla (SNA 83°) and mandible (SNB 79°) with mildly proclined upper (U1-NA 26°) and lower anteriors (L1-NA 28°) (IMPA 96°). The FMA value of 27° and Go-Gn 32° showed that the patient had a normal growth pattern (Figure 2, Table 1).

**Table 1:** Pre-cephalometric values

Parameters	Pre-treatment
SNA	83°
SNB	79°
ANB	4°
U1- NA	26°
L1-NB	28°
Go-Gn- SN	32°
FMA	27°
IMPA	96°
G-Sn-Pg	7°
Nasolabial angle	105°

Treatment plan

The ideal treatment plan included surgical commissuroplasty, restoration of the carious tooth 46, extraction of the ectopically erupted 23, and removal of the lingually displaced mandibular second premolars. However, the patient declined both commissuroplasty and extraction of the impacted teeth (35 and 45) because of restricted mouth opening and unwillingness to undergo surgery. Accordingly, an alternative plan was implemented, which involved the extraction of tooth 23, followed by leveling and alignment. Fixed mechanotherapy was carried out using a pre-adjusted edgewise appliance with a 0.022” slot. Alignment and leveling were initiated with Ni-Ti wires, while torque corrections were achieved using stainless steel archwires. The primary goal was to establish proper overjet and overbite, followed by finishing and settling to enhance stability. Retention was ensured through a combination of a fixed lingual retainer and a removable retainer designed for ease of use by the patient.

Treatment procedure

**Figure 3:**
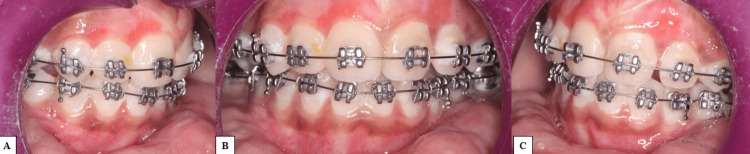
Maxillary and mandibular arch post bonding A. Right quadrant. B. Frontal intraoral. C. Left quadrant.

The patient was referred for necessary restorative and extraction procedures before initiating orthodontic treatment. However, the parents declined the extraction of the lingually rolled mandibular premolars. Oral prophylaxis was carried out prior to the procedure. The molars were banded with preformed bands using glass ionomer cement, and bonding was performed with pre-adjusted edgewise MBT prescription brackets (0.022 × 0.025 inch slot) in both arches. Due to limited mouth opening and difficulty in isolation, bonding was done two teeth at a time. The archwire sequence consisted of 0.013 CuNiTi and 0.014 Ni-Ti wires for initial alignment over four months, followed by 16 × 22 Ni-Ti and 17 × 25 CuNiTi for the next four months, and 17 × 25 stainless steel for one month (Figure 3).

**Figure 4:**
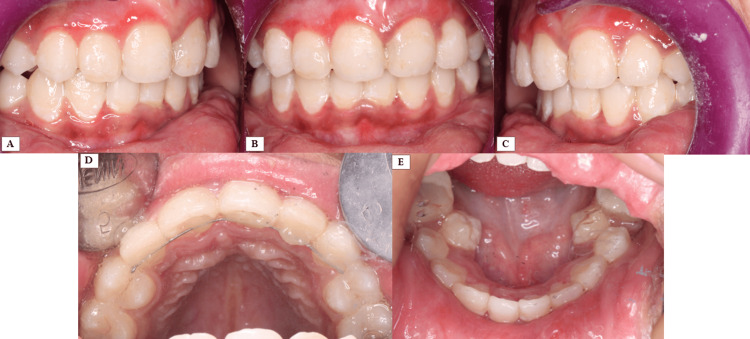
Post-treatment intraoral photographs A. Right quadrant. B. Frontal intraoral. C. Left quadrant. D. Maxillary occlusal. E. Mandibular occlusal.

**Figure 5:**
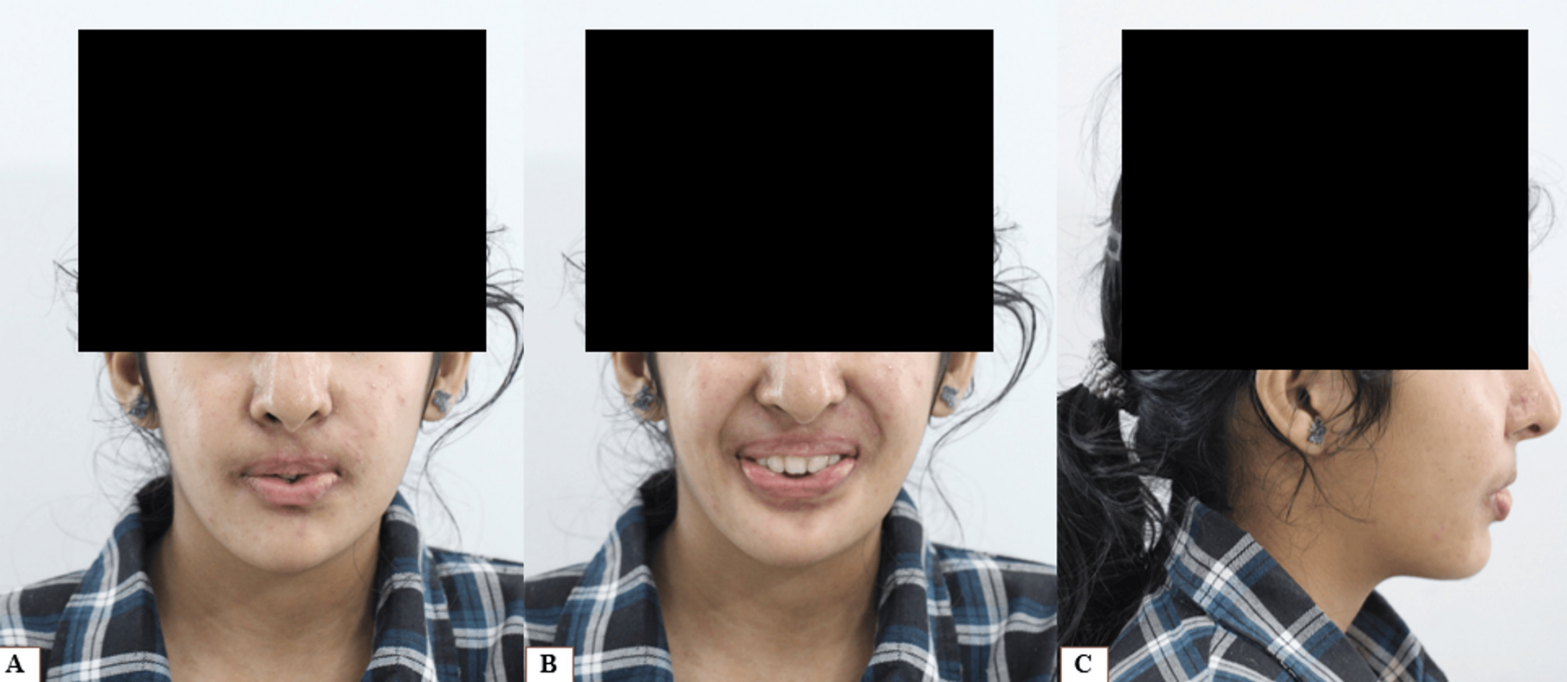
Post-treatment extraoral photographs A. Frontal extraoral. B. Frontal extraoral with smile. C. Profile extraoral.

The patient maintained excellent compliance with minimal bracket failures during treatment. After achieving adequate leveling and alignment, both arches were debonded, and a fixed lingual retainer was placed in the maxillary arch (Figures 4, 5). Post-treatment, a digital scan was obtained for fabrication of the removable retention appliance.

Retention protocol

**Figure 6:**
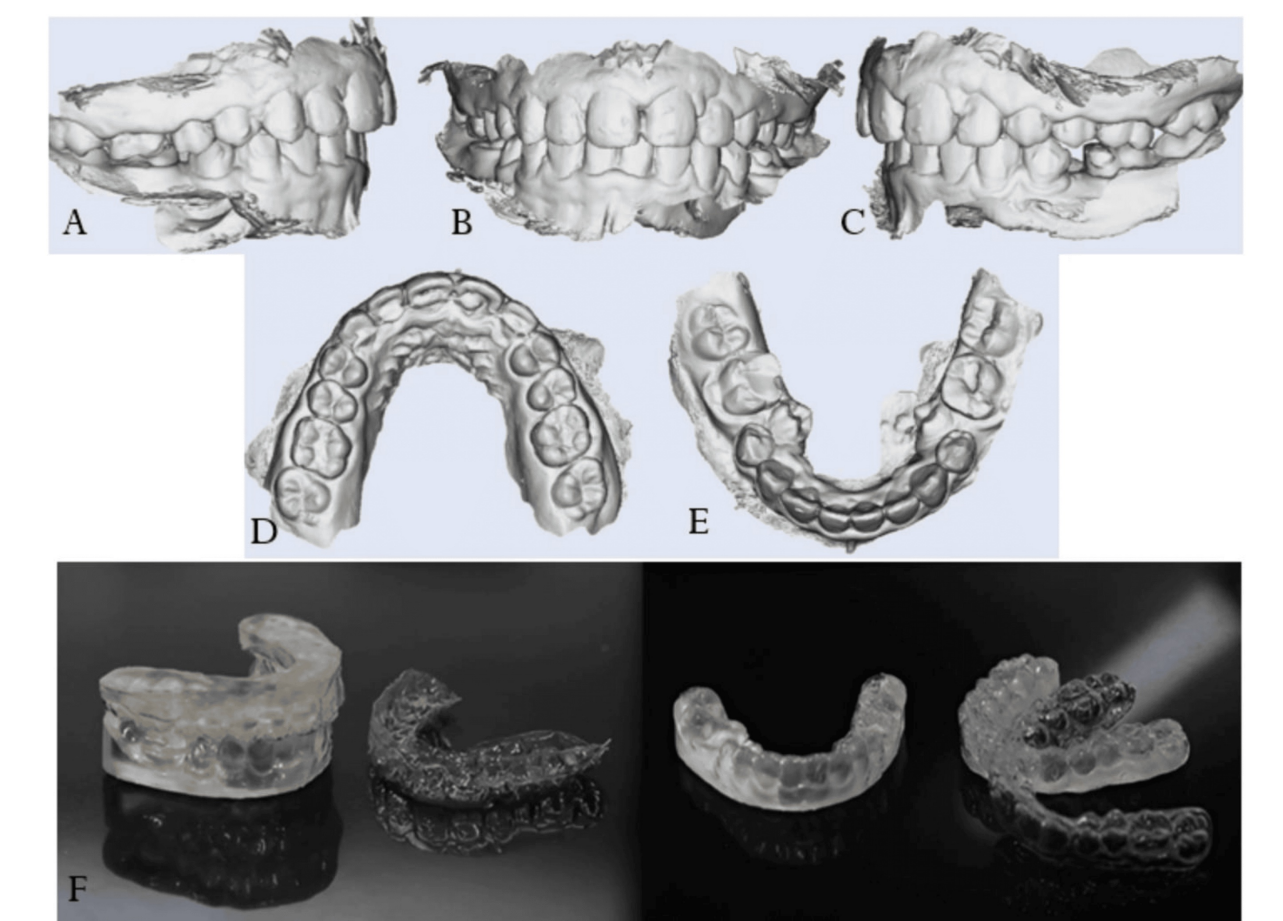
A. Right quadrant. B. Frontal intraoral. C. Left quadrant. D. Maxillary arch occlual. E. Mandibular arch occlusal. F. Thermoformed Essix retainers.

The post-treatment intraoral scan was modified to preserve only the anterior one-third of the palate, ensuring stability of the achieved transverse dimensions. The digital scan was then used for printing a 3D model of the patient’s upper and lower arches using a medical-grade resin. Essix retainer sheets were thermoformed onto these 3D models. The retainers were cut 2 mm above the gingival margin, while the anterior third of the palatal surface was kept intact. The patient was instructed to wear the retainers full-time, except during meals and oral hygiene practices, and was scheduled for follow-up visits at six-month intervals( Figure 6).

Results

**Figure 7:**
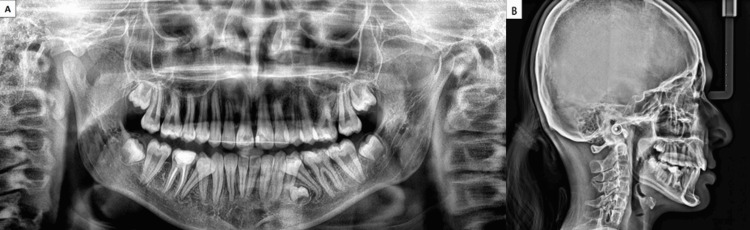
A. Postoperative orthopantomogram. B. Postoperative lateral cephalogram.

Post-treatment clinical assessment indicated that by the end of therapy, an ideal overjet and overbite were established, and the Class I molar relationship was maintained. The upper and lower anterior crowding was considerably reduced except for the lingually rolled-in second premolars, which were planned for extraction. The upper midline was shifted to the left as the canine had to be extracted. The post-treatment cephalometric evaluation demonstrated a reduction in the proclination of both maxillary and mandibular incisors (Table 2, Figure 7). The interincisal angle (U1-L1) came up to normal post-treatment, with a value of 130°.

**Table 2:** Post-cephalometric values

Parameters	Post-treatment
SNA	83°
SNB	79°
ANB	4°
U1- NA	23°
L1-NB	17°
Go-Gn-SN	32°
FMA	27°
IMPA	81°
G-Sn-Pg	16°
Nasolabial angle	98°

## Discussion

FSS, also known as Freeman-Burian syndrome, is a rare congenital disorder characterized by craniofacial and musculoskeletal abnormalities. Dental treatment was compromised in most cases reporting with FSS, the major reason being microstomia and restricted mouth opening [4].

Earlier case reports, such as that by Olkun et al. (2019), described the management of a 16-year-old patient using a Forsus appliance followed by fixed orthodontic therapy [5]. Vyas et al. [6] had suggested surgical intervention, including commissuroplasty, for the correction of microstomia; however, in the present case, this option was avoided as the patient was not willing to undergo surgery. Instead, fixed mechanotherapy with pre-adjusted edgewise appliances was employed, which resulted in satisfactory arch expansion and alignment of the dentition.

The current literature evidence offers no standardized treatment protocol for managing patients with FSS, as the clinical presentation varies considerably among individuals. In the present case, the patient’s chief concerns were managed successfully through a non-surgical approach. Levelling and alignment of both maxillary and mandibular arches were accomplished using the pre-adjusted edgewise technique. However, long-term post-retention stability remains uncertain due to the strong perioral musculature exerting excessive forces on the dentition. Therefore, careful planning of the retention phase is essential to preserve the corrections achieved. The use of Essix retainers with adequate thickness, extending over at least the anterior one-third of the hard palate, is recommended to ensure stability of the expansion gained through fixed appliance therapy.

## Conclusions

Orthodontic treatment of FSS poses challenges due to microstomia and limited oral access. This case report highlights that satisfactory alignment can be achieved through a non-surgical fixed mechanotherapy with a pre-adjusted edgewise appliance. Long-term stability, however, relies heavily on an appropriate retention strategy, with Essix retainers and fixed lingual retainers playing a key role in maintaining the corrections achieved.
